# Association of migration and family planning use among women in Malawi: Evidence from 2019/2020 Malawi Multiple Indicators Survey

**DOI:** 10.1186/s40834-023-00254-8

**Published:** 2023-10-27

**Authors:** Reuben Christopher Moyo, Dumisani Nkhoma

**Affiliations:** 1 Division of Epidemiology and Biostatistics, Faculty of Medicine and Health Sciences, Ministry of Health, Nkhatabay District Health Office, Stellenbosch University, Nkhatabay, Cape Town, Malawi; 2 Christian Health Association of Malawi, Lilongwe, Malawi

**Keywords:** Malawi, Contraceptive prevalence rate, And Migration

## Abstract

**Background:**

Family planning (FP) is known to bring multiple benefits to people both individually and collectively. Individually, FP has been associated with reduction in risk of unintended pregnancy which also correlates with low child mortality rates. Child mortality rates in women with child spacing of less than two years are 45% higher compared to their counterparts with child spacing of more than two years. Several factors that predict FP utilisation among women of childbearing age have been identified but there is limited literature on how migration impacts FP utilisation in Malawi. Our current study aimed at assessing the association between migration and modern contraceptive use among women of childbearing age in Malawi.

**Methods:**

Data for this study came from a nationally representative 2019/20 Malawi multiple cluster indicator survey (MICS). At total of 24,543 women aged 15 to 49 participated in the survey. Contraceptive prevalence rate (CPR) analyses were conducted separately on all women of childbearing age and married women. The data was analysed using the complex survey data approach by applying sampling weights to correct unequal representation of participants at cluster, district, and regional level. We used binary logistic regression to assess association between migration status and modern contraceptive use among all women of childbearing age and married women separately. We included age, age at first sex, age at marriage, region of residence, education, residence wealth index and presence of disability as confounders in our final multivariable models.

**Results:**

The overall CPRs for married women and for all women of childbearing age were 64.7% and 40.5% respectively. The CPRs for all women of childbearing age were 40.5% for non-migrants and 33.0% for migrant women. For married women, CPRs were 51.5% for migrant women and 65.5% for non-migrant women. The fully adjusted odds ratios for the association between migration status and modern contraceptive use were 0.62 (0.49–0.78) for married women and 0.65 (0.52–0.80) for all women of childbearing age.

**Conclusions:**

We conclude from our findings that migrant women were significantly less likely to utilize modern contraceptive methods for both married women and all women of childbearing age. Deliberate efforts are required to ensure that migrant women of childbearing age have equal access to sexual and reproductive health services which includes family planning.

## Background

Family planning (FP) is known to bring multiple benefits to people both individually and collectively. Individually, FP has been associated with reduction in risk of unintended pregnancy which also correlates with low rates of Human Immuno-deficiency Virus (HIV) mother to child transmission (MTCT) rates [1]. Child mortality rates among women with child spacing of less than two years are 45% higher compared to their counterparts with child spacing of more than two years [2]. Women using modern FP methods are more empowered compared to women not using modern contraceptive methods. Collectively, FP has been associated with positive increase in economic and educational outcomes [2].

FP progress is usually measured through several indicators namely, contraceptive prevalence rate (CPR), unmet need, fertility rate and the novel demand for modern family planning methods satisfied (mDFPs). CPR is the percentage of women who are currently using, or whose sexual partner is currently using, at least one method of contraception, regardless of the method used usually reported for women in the reproductive age group (15–49 years) who are in union or married [1]. Unmet need represents women, fecund and sexually active, who report not using any contraceptive method and report no desire for more children or at least want to delay further pregnancies [1]. The mDFPs on other hand is defined as the proportion of women in the reproductive age group, who desire no further or wish to delay pregnancy and are using modern contraceptives [3]. Finally, total fertility rate is the number of children a woman could have in her reproductive period in accordance with the age-specific fertility rates in that specific year [4]. CPR and unmet need were used to measure FP progress during the millennium development goals (MDGs) era and are still relevant to date. The mDFPs was introduced as a measure during the sustainable development goals (SDGs) era.

Globally, 1.1 billion women report a need for use of contraception, of which 270 million have an unmet need [2]. Out of these 270 million women, about 214 million reside in developing countries. The mDFPs is currently estimated at 77% globally, and 58% in Africa [5]. CPR by any method in all women and in women in the reproductive age group are estimated to be 49% and 63% respectively. Total fertility rate globally is estimated at 2.4 per woman [5]. In Malawi, CPR by modern method and unmet need stands at 64.7% and 26% for women in union or currently married, which is roughly comparable to international standards [6]. A recent study on mDFPs estimated that mDFPs for Malawi was approximately 74% [7]. Total fertility rates dropped from 5.7 to 2010 to 4.2 per woman in 2018 [8]. Such progress has been made possible in part due to policy instruments such as the sexual and reproductive health and rights (SRHR) strategy 2017–2022 document and global commitments such as the FP2030 partnerships [9, 10]. FP indicators vary widely and are affected by several socio-demographic and health system characteristics [7]. For example, contraception use has been known to vary by region, religion, income status, number of children ever born, access to information and availability of proximate FP services [7]. Noting such variations is important for policymakers and major players in the sexual and reproductive health (SRH) sector as the nation pushes to improve SRH outcomes.

Migrants are estimated to account for about one billion globally of which approximately 48% are women of childbearing age [11]. Migration as a predictor of modern contraceptive use is rarely investigated in many countries including Malawi. Studies on factors that predict modern contraceptive use in SSA have reported consistent findings on predictors of modern contraceptive use and these are age of the participants, low education levels, poverty, children ever born, age at first sex, married marital status, less exposure to media, rural residence, antenatal visits, and delivery at a health facility [12–14]. Very few studies in SSA, Africa and the rest of the world have investigated the impact of migration status on modern contraceptive use [11, 15–20]. Most studies that investigated the impact of migration on contraceptive use found that migrants have unequal access to modern contraceptive compared to their counterparts.

Comprehending contraception use and access among migrants is vital for a couple of reasons. Firstly, migrants tend not to fare well in other health outcomes. For example, a study in Malawi showed a high HIV prevalence among migrants [21]. Secondly, FP research among immigrants has given contradicting results with many studies reporting limited access to modern contraceptives among migrant. A study in Zambia in 2019 found high unmet need for family planning among rural to rural migrants [11]. Studies in Europe showed high abortion rates and low contraception use among immigrants compared to non-immigrants [22, 23]. Another study of Eritrean refugees in Ethiopia showed an unmet need of 41% among migrant women of childbearing age [24]. On the other hand, similar studies in Nigeria and Kenya found higher use of modern contraceptive among internal migrants [15, 25]. In Ghana, a study on contraceptive use among adolescent migrants found that only 13% of adolescent girls were using contraceptive methods to delay pregnancy [26]. In Vietnam, a recent study on access to SRH services by migrant women found that AGYW accessed SRH the least compared to older women [27].Alternatively, such variations might also be influenced by the type of migration involved and differences in setting and density of migration. The impact of migration on modern contraceptive use has received little attention as there is limited literature on its in Malawi and other African Countries.

FP is essential for achieving sexual and reproductive health rights and developmental goals of women of childbearing age and the entire population [28]. Thus, it is essential that modern contraceptive methods are accessible to all groups of people, including migrants in line with SDG 3.8 which emphasizes universal health coverage and 3.7.1 which specifically targets universal access to SRH services [3]. Migration is described as the movement of persons away from their place of residence, either across an international border or within a state [29, 30]. The United Nations (UN) defines short term migrant as “a person who has changed residence for a period of at least 3 months, but less than a year (12 months) except in cases where the movement is for purposes of recreation, holiday, visits to friends or relatives, business, medical treatment or religious pilgrimage [30]. Migration is an important factor in health and development as it affects population dynamics of a country [31]. This study therefore aimed at assessing association between migration status and modern contraceptive use among women of childbearing age in Malawi.

## Methods

### Study setting, data sources and population

Malawi is a landlocked country with an estimated area of 118,484 km^2^ and a 2023 projected population of 20.6 million [8]. Urban centers hold approximately 16% of the population [8].

We used a cross-sectional study design using the 2019/20 Malawi Multiple Indicator Cluster Survey (MICS). MICS is a nationally representative household survey that provides up-to-date information on sexual behavior, child health indicators, HIV, maternal health, fertility, childbearing as well as hygiene and sanitation. MICS are conducted every four to five years by the Malawi national statistics office (NSO) and Ministry of Health with technical support from the United Nations Children’s Funds (UNICEF). Details of the sampling strategy and methodology of the MICS have been described in detail elsewhere [6]. A total of 25,543 women aged 15 to 49 were asked to participate in the survey but 24,543 women participated in the survey.

### Outcome, exposure and confounder variables

#### Outcome variable

The outcome variable was any modern contraceptive use among women of childbearing age (aged 15 to 49 years). Modern family planning use was coded 1 for currently using any modern contraceptive and 0 for not using. Modern contraceptive use was restricted to use of the following methods: female sterilization, male sterilization, IUD, injectable, implants, pills, male condom, Female condom, diaphragm, foam, jelly and lactation amenorrhea (LAM). This variable was created by recoding 1 for use of each of the above method and 0 for not using each of the above method. The overall CPR variable was generated by generating a variable using the egen rowmax command which created a variable coded 1 for any use of the above-mentioned methods and 0 for not using any method. Use of modern contraceptive was assessed by asking women if they were currently using any family planning method mentioned above. Those who responded with a yes were further asked about the method they were using to avoid pregnancy. Any method indicated above was considered modern contraceptive method.

#### Main exposure/ predictor variable

The main exposure was migration status. This variable described whether the participant changed residence within 12 months, coded 1 for women who recently changed residence within 12 months and 0 for women who did not change residence regardless of the type of migration involved (rural-rural, rural-urban, urban-rural as well as urban-urban). Migration status variable was generated from the duration of stay at current residence variable that ranged from 0 to 49 years by recoding 0 (representing women who changed residence for less than 12 months) into 1 representing migrants and ≥1 (representing women who did not change residence within 12 months) into 0 representing non-migrants. This definition of migration was in accordance with the United Nations definition of short-term migration [26].

#### Confounding variables

Covariates that were identified within the 2019/20 MICS dataset as potential confounders were participants age group (categorized into 15 to 19, 20 to 24, 25 to 29, 30 to 34, 35 to 39, 40 to 44 and 45 to 49), age at first sex (categorized into ≤15, 16 to 19 and ≥ 20), age at first marriage (categorized into ≤15, 16 to 19 and ≥ 20), marital status (categorised into married, formerly married and never married), residence (rural versus urban), region (northern, central and southern region), participants wealth index (categorized into lowest, second, middle, fourth and highest), education (categorized into pre-primary, primary, lower secondary, upper secondary, higher and vocational training), any functional disability (categorized as having any functional disability and not having) and children ever born (categorized into 0, 1 to 2, 3 to 5 and ≥ 6).

### Statistical analysis

Data for this study was downloaded from MICS website (https://mics.unicef.org) in SPSS format and was exported into Stata 18.0 [32] for analyses. After data cleaning, we declared the data as survey data with complex design features using svyset command to allow use of sampling weights to correct unequal representation of participants at cluster, district, and regional level. All subsequent analyses utilised the svy prefix for survey data analysis. Results were stratified by married women (N = 14,934) and all women of childbearing age (N = 24,543). We grouped and recoded some variables that had more numeral values for ease of analysis and comparison such as age at first sex, age at first marriage, age and duration of residence. For variables on use of specific FP method, women who indicated they were using a method were coded 1 and those not using that particular method were recoded 0 indicating they were not using that particular method. These variables were grouped based on common reporting of these variables in literature.

To examine association between any modern contraceptive use and potential confounders one at a time, Pearson’s chi-square tests (X^2^) were conducted. Predictors were considered significant at p < 0.05. We investigated for possible multicollinearity among independent variables using pairwise correlation. Variables with correlation coefficient (r) ≥ 0.5 either direction were not included in the final multivariable model.

We estimated two separate binary logistic regression models for married women and for all women of childbearing age. Children ever born and marital status were not included in the final models due to their strong linear relationship with women’s age. The variables included in the final multivariable models had missing observation on age at first marriage, age at first sex and migration status. The missing values were only observed on all women of childbearing dataset. We performed multiple imputation using chained equations (MICE) to impute the missing observations [31]. Consequently, estimation of the final logistic regression model was performed using multiple imputation with Monte Carlo error estimates for odds ratios to assess association of migration status and modern contraceptive use among women of childbearing age, controlling for the independent effects of other confounders. The final model had participants age, age at first sex, age at first marriage, residence, region, levels of education, and wealth index as confounders.

## Results

### Socio-demographic characteristics of all women of childbearing age

Table 1 shows distribution of selected socio-demographic characteristics of all women of childbearing age. A total of 24,543 women aged 15–49 years participated in the MICS. 81.8% of the participants came from rural areas while18.2% from urban areas. The weighted proportions of women representing the central, southern and northern regions were 45.1%, 43.4% and 11.1% respectively. Approximately 21% of the women reported having started sex before their fifteenth birthday and nearly the same proportion got married by their fifteenth birthday. 77% of the participants had no secondary education while only 2.4% had higher education. 62% of the women were currently married or living with a partner compared to 22% who were not in union. The proportion of women who reported changing residence within 12 months accounted for 5.3%.

**Table 1:** Socio-demographic characteristics of the women (N = 24,543)

Characteristic	Frequency	Weighted Col Percentage
**Age Groups**		
15–19	5,770	22.61
20–24	4,697	19.47
25–29	3,864	16.02
30–34	3,249	13.83
35–39	3,024	12.42
40–44	2,270	08.97
45–49	1,669	06.69
**Migration status**		
Yes	1,279	5.27
No	23, 264	94,73
Residence		
Rural	20,486	81.84
Urban	4,057	18.16
**Region**		
Northern	5,348	11.11
Central	8,075	45.51
Southern	11,128	43.39
**Age at first sexual intercourse (years)**		
< = 15	5,270	21.03
16–19	10,891	45.68
20 and above	5,124	20.44
Never had sexual intercourse	3,257	12.85
**Marital Status**		
Currently married/in union	14,934	62.10
Formerly married/in union	3,800	15.54
Never married/in union	5,809	22.36
**Age at first marriage or union (N = 18,734)**		
< =15	3,857	20.40
16–20	11,514	62.12
21–25	3,363	17.48
**Education level**		
Pre-primary or None	2,014	08.76
Primary	15,662	64.08
Lower Secondary	3,061	11.80
Upper Secondary	3,245	12.28
Higher	505	02.22
Vocation training	56	0.28
**Wealth Index**		
Poorest	4,402	20.00
Second	4,476	18.97
Third	4,828	18.86
Fourth	5,172	19.62
Richest	5,665	22.55
**Any Functional Disability**		
Yes	1,163	4.37
No	19,961	82.41
Missing	3,419	13.22
**Number of Children Ever Born**		
Zero	5,823	23.02
1–2	4,197	16.92
3–5	3,539	14.65
6 and above	10,984	45.42

### Comparison of modern contraceptive use by married women and all women of childbearing age

Table 2 shows comparison of CPRs for married women and for all women of childbearing age by selected socio-demographic characteristics. The overall CPR among married women was 64.7% while that of all women of childbearing age was 40.2%. CPRs for women in union were 51.6% for migrant women and 65.5% for non-migrant women. CPRs among all women of childbearing age were 33.0% for migrant women and 40.6% for non-migrant women of childbearing. CPR was slightly higher in the central region (68.9% for women in union and 44.3 for all women of childbearing age) compared to southern region (61.5% for married women and 36.7% for all women of childbearing age) and northern region (59.7% for married women and 37% for all women of childbearing age). CPR for participants who started sex before their fifteenth birthday was 65.5% for married women and 43.3% for all women of childbearing age. Women with higher education had low CPR for both married women (56%) and all women of childbearing age (26%) compared to women with low education levels. Compared to women aged over 20 years, women aged 15 to 19 had low CPR (46.5% for married women and 9.71% for all women of childbearing age). Results from Table 2 further shows there were no significant differentials in CPR by women’s wealth index, residence, and functional disability for both married women and all women of childbearing age. Factors associated with modern contraceptive use for both married women and all women of childbearing age were migration status (p < 0.001), participant’s age (p < 0.001) age at first sex (p < 0.001), age at first marriage (p < 0.0159), region (p < 0.001) and levels of education (p = 0.023).

**Table 2:** Comparison of modern family planning use among participants by selected characteristics

	Currently using any modern method of contraception					
Predictors	Women in union or married (N = 14,934)			All Women of child bearing age (N = 24,543)		
	Yes (Row %)	No (Row%)	P-Value	Yes (Row %)	No (Row%)	P-Value
Overall	9,527 (64.74)	5,407 (35.26)		9,527 (40.20)	15,016 (59.80)	
**Migration status**						
Yes	386 (51.45)	401 (48.55)	< 0.001	386 (32.98)	893 (67.02)	< 0.001
No	9,140 (65.50)	5,006 (34.50)		9,141 (40.61)	14,123 (59.39)	
**Age Group (years)**						
15–19	564 (46.50)	604 (53.50)	< 0.001	564 (09.71)	5,206 (90.29)	< 0.001
20–24	1,974 (65.22)	1,075 (34.75)		1,974 (43.28)	2,723 (56.72)	
25–29	2,024 (68.35)	977 (31.65)		2,024 (53.55)	1,840 (46.45)	
30–34	1,770 (70.07)	787 (29.93)		1,770 (55.43)	1,479 (44.57)	
35–39	1,545 (68.32)	755 (31.68)		1,545 (52.86)	1,479 (47.14)	
40–44	1,058 (64.38)	623 (35.62)		1,058 (48.32)	1,212 (51.68)	
40–49	592 (52.58)	586 (47.42)		592 (36.47)	1,077 (63.53)	
**Residence**						
Urban	1,384 (64.52)	802 (35.48)	0.8942	1,384 (36.42)	2,673 (63.58)	0.006
Rural	8,143 (64.78)	4,605 (35.22)		8,143 (41.04)	12,343 (58.96)	
**Region**						
Northern	1,933 (59.66)	1,237 (40.34)	< 0.001	1,933 (37.15)	3,415 (62.85)	< 0.001
Central	3,417 (68.82)	1,687 (31.18)		3,417 (44.29)	4,658 (55.71)	
Southern	4,176 (61.46)	2,483 (38.53)		4,177 (36.70)	6,943 (63.30)	
**Age at first sexual intercourse (years)**						
≤15	2,136 (65.57)	1,227 (34.43)	< 0.001	2,136 (43.26)	3,134 (56.74)	< 0.001
16–19	4,874(64.75)	2,758 (35.24)		4,875 (45.84)	6,016 (54.16)	
20 and above	2,516 (63.95)	1,422 (36.05)		2,516 (49.73)	2,608 (50.27)	
**Marital Status**						
Married /In Union	9,527 (64.74)	5,407 (35.26)	< 0.001	9,527 (64.74)	5,407 (35.26)	< 0.001
Formerly Married	N/A	N/A		0 (0.00)	3,800 (100.0)	
Never Married	N/A	N/A		0 (0.00)	5,808 (100.0)	
**Age at Marriage**						
≤15	1,941 (65.50)	1,064 (35.50)	0.028	1,941 (51.34)	1,916 (48.66)	0.0178
16–19	5,991 (65.40)	3,303 (34.60)		5,991 (52.74)	5,523 (47.26)	
>= 20	1,595 (61.44)	1,040 (38.56)		1,595 (48.88)	1,768 (51.12)	
Not Married	N/A	N/A		0 (0.00)	5,809 (100.0)	
**Education level**						
None	903 (61.7)	573 (38.31)	0.007	903 (45.80)	1,111 (54.21)	< 0.001
Primary	6,408 (65.10)	3,579 (34.90)		6,408 (42.22)	9,254 ( 57.78)	
Lower Secondary	1,064 (66.68)	569 (33.32)		1,064 (36.05)	1,997 (63.95)	
Upper Secondary	1,000 (65.60)	567 (34.37)		1,000 (32.2)	2,245 ( 67.08)	
Higher	141 (56.11)	104 (43.89)		141(26.02)	364 (72.57)	
Vocation Training	11 (32.45)	15 (67.55)		11 (14.65)	45 (85.35)	
**Wealth index quintile**						
Poorest	1,729 (65.78)	947 (34.22)	0.1273	1,729 (40.50)	2,673 (59.50)	< 0.001
Second	1,855 (65.63)	1,056 (34.37)		1,855 (43.33)	2,621 (56.67)	
Third	1,988 (65.98)	1,044 (34.02)		1,899 (41.49)	2,840 (58.51)	
Fourth	1,989 (62.19)	1,189 (37.81)		1,989 (40.14)	3,183 (59.86)	
Richest	1,966 (64.22)	1,171 (35.78)		1,966 (36.28)	3,699 (63.72)	
**Any Disability**						
Yes	491 (64.84)	301 (35.16)	0.9594	491 (43.22)	672 (56.78)	< 0.001
No	8,973 (65.10)	4965 (34.90)		8,971(46.24)	10,988 (53.76)	
Missing	63 (31.62)	141 (68.38)		63 (01.58)	3,356 (98.42)	

### Association of migration status and modern contraceptive use among married women and all women of childbearing

Table 3 shows results from a multivariable logistic regression model of the association between migration status and modern contraceptive use among married women and all women of childbearing age. The crude odds ratio (COR) for the association between migration status and modern contraceptive use was 0.55 (95% CI 0.47–0.63, p < 0.001) for married women and 0.72 (95% CI 0.53–0.84, p < 0.001) for all women of childbearing age. After controlling for the effects of participants age, age at first sex, age at first marriage, levels of education, residence, region, wealth index and presence of disability the adjusted odds ratio (AOR) for the association between migration status and modern contraceptive use was 0.61 (95% CI 0.49–0.77, p < 0.001) for married women and 0.65 (95% CI 0.52–0.80, p < 0.001) for all women of childbearing age. Results from Table 3 further shows that the odds of using modern contraceptive methods increased with increase in age but decreased among women aged 40 and above for both married women and all women of childbearing age. Women from the central region had slightly higher odds of utilizing modern FP methods for all categories of women compared to women from the southern and northern regions. Results further show no significant differentials in odds of modern contraception use by wealth index, level of education, age at marriage and age at sexual debut for both married women and all women of childbearing age.

**Table 3:** Association between migration and modern contraceptive use among married women and all women of childbearing age

	In Union/Married Women		All Women of Childbearing age	
Predictor	**OR (95% CI)**	**P-Value**	**OR (95% CI)**	**P-Value**
Crude Odds Ratio	**0.55 (0.50–0.78)**	**< 0.001**	**0.72 (0.53–0.84)**	**< 0.001**
**Migration status**				
No	1			
Yes	0.61 (0.49–0.77)	< 0.001	0.65 (0.52–0.80)	< 0.001
**Age group**				
15–19	1		1	
20–24	1.93 (1.54–2.40)	< 0.001	1.54 (1.27–1.88)	< 0.001
25–29	2.27 (1.85–2.77)_	< 0.001	1.69 (1.40–2.04)	< 0.001
30–34	2.48 (2.04–3.03)	< 0.001	1.64 (1.37–1.98)	< 0.001
35–39	2.30 (1.85–2.85)	< 0.001	1.47 (1.22–1.76)	< 0.001
40–44	1.97 (1.57–2.48)	< 0.001	1.19 (0.97–1.46)	0.098
45–49	1.24 (0.97–1.59)	0.076	0.74 (0.59–0.92)	0.008
**Age at first sexual intercourse (years)**				
≤ 15	1		1	
16–19	0.90 (0.79–1.07)	0.089	0.96 (0.86–1.08)	0.518
≥20	0.85 (0.73–0.99)	0.042	0.99 (0.87–1.12)	0.846
**Age at First Marriage**				
≤ 15	1		1	
16–19	1.01 (0.87–1.18)	0.912	1.02 (0.91–1.15)	0.781
≥ 20	0.81 (0.68–0.96)	0.016	0.88 (0.76–1.01)	0.068
**Levels of Education**				
No Education	1		1	
Primary Education	1.14 (0.99–1.32)	0.066	1.06 (0.94–1.19)	0.355
Lower Secondary	1.27 (1.03–1.58)	0.024	1.00 (0.86–1.18)	0.959
Higher Secondary	1.27 (0,98–1.63)	0.060	1.01 (0.83–1.24)	0.917
Higher Education	0.93 (0.58–1.48)	0.755	0.83 (0.58–1.19	0.313
Vocation Training	0.33 (0.13–0.87)	0.024	0.32 (0.12–0.75)	0.010
**Residence**				
Urban	1		1	
Rural	0.99 (0.83–1.20)	0.998	1.12 (0.96–1.33)	0.155
**Region**				
Northern	1		1	
Central	1.47 (1.25–1.70)	< 0.001	1.43 (1.24–1.65)	< 0.001
Southern	1.06 (0.92–1.230	0.426	0.96 (0.84–1.02)	0.591
**Wealth Quintile**				
Poorest	1		1	
Second	0.98 (0.85–1.13)	0.824	1.26 (1.12–1.44)	< 0.001
Third	1.03 (0.86–1.19)	0.737	1.36 (1.20–1.54)	< 0.001
Fourth	0.85 (0.73–0.97)	0.019	1.3(1.20–1.53)	< 0.001
Richest	0.94 (0.77–1.14)	0.507	1.56 (1.31–1.84)	< 0.001
Any Disabilities				
Yes	1		1	
No	0.98 (0.79–1.19)	0.790	1.11 (0.95–1.82)	0.188

## Discussion

This study aimed at assessing association between migration status and modern contraceptive use among women of childbearing age in Malawi. Our results have shown that migrant women have significantly low CPR and are probably less likely to access modern contraceptive methods for both married women and all women of childbearing age compared to non-migrants.

Our findings contradict a study that found that migrant women had higher modern family planning utilisation rates compared to non-migrants in Kenya in 2016 [18] and in Nigeria in 2018 [25]. Our findings are consistent with a related study conducted in India in 2014 on differentials in utilisation of modern contraceptives by migration status as CPR for migrants was 40% while that of non-migrants was 48% [33]. In line with our findings is a study on type of migration on modern contraceptive use in Myanmar in 2019 that found rural to rural migrants had low access to contraceptive methods compared to urban to urban as well as rural to urban migrants [16]. A slightly different study on association between women left behind by men who have migrated and modern contraceptive use in India found that women left behind by migrant men had low CPR compared to their counterparts [19]. Results from a number of studies reviewed on the impact of migration on modern contraceptive use have reported low utilization of FP methods among migrant women apart from the two studies with inconsistent results. While study setting may explain variations on the impact of migration on modern contraceptive use, a study on the impact of internal migration on unmet need for modern contraceptive in Zambia (a neighboring country to Malawi) found high unmet need for contraception among internal migrants [11].Our findings therefore support evidence found in many other studies and countries that found that migration significantly impacts on reproductive health choices among women of childbearing age. Some of the possible reasons for the association found between migration status and low utilisation of modern contraceptive by migrant women could be attributed to limited information on availability of SRHR services in their new environments, legal restrictions for international migrants who do not have permits, language barriers, competing priorities [34], spousal separation [17], fear of side effects, and cultural or religious reasons [35].

While the type of migration may help explain the findings better, this study did not categorize type of migration involved (rural to urban, urban to rural, urban to urban as well as rural to rural) as this classification was not available in MICS dataset. The differences in findings from studies that reported inconsistent results could be attributed to study setting, density of migration as well as its classification (rural-urban, urban-rural, rural-rural, and urban-urban).

Amongst women at risk of migrating are adolescent girls and young women (AGYW) who move from rural areas to urban areas and vice-versa for various social and economic reasons [36, 37]. AGYW in Malawi have also been shown to have high childbearing rates but their corresponding CPR as found in this study are low compared to older women [38]. Low contraceptive use among AGYW including those migrating is likely to frustrate the fight against teenage childbearing in Malawi is one of the leading causes of high fertility rates, unsafe abortions, and unwanted childbearing. Our findings are consistent with recent studies on access to sexual and reproductive health services among female migrants in Vietnam and Ghana that found that AGYW had low access to SRH services including family planning [26, 27]. Some of the contributing factors to low CPR among AGYW are limited access to information on modern family planning methods, myths and misconceptions surrounding use of contraceptive methods such as failure to regain fertility, unavailability of emergency contraceptives in some facilities as well as unfriendly or unresponsive youth friendly services [39, 40]. A study on barriers and motivators of contraceptive use among AGYW in Malawi found that supportive social networks, respect for privacy and confidentiality, availability of commodities, affordability of services, accessibility of contraceptives and desire to prevent unintended pregnancy and sexually transmitted infections were the motivators of contraceptive use among young people in Malawi [41]. Myths and misconceptions, known side effects of contraceptives, prohibitive social norms, and negative attitude of health professionals are some of the barriers to contraceptive use among AGYW in SSA [42]. Efforts to increase CPR for AGYW include but not limited to targeted outreach clinics to target underserved populations as well as areas mostly accessed by young people including migrant camps, provision of adolescent friendly and responsive services at facility and community level, making all modern contraceptive readily available in facilities including emergency contraceptives as well as intensifying provision of modern contraceptives through community-based distribution agents of modern contraceptives.

While the influence of both internal and external migration on access to SRH services receive little attention in most SSA countries, its impact on the reproductive health choices and its associated rights is well documented [17, 43, 44]. Ensuring that migrant women have equal access and sensitisation to modern contraceptive and other SRH services is important for equality of the fulfillment of SRH rights. Failure to meet the family planning needs of the migrating women may result in increasing their unmet need for modern family planning as well as contributing to high fertility rates that may impact on the population dynamics of the nation. Deliberate strategies for ensuring that sexual and reproductive health rights of all migrant women are required for all types of migrants in order to improve their SRH choices.

Our study is strengthened by its nationally representative large sample size and is the first of its kind in Malawi to examine the impact of migration on CPR among women of childbearing age in Malawi. Our findings have both policy applications and implications for both policy makers and program managers in the SRH sphere in Malawi. Firstly, results could be used to design strategies of reaching both internal and international migrants with SRH services including family planning to improve their SRH outcomes. Secondly, our findings may be used by organizations working with migrants to evaluate their strategies of reaching out to migrants with SRH services based on the identified correlates of contraceptive use to ensure that no one is left behind. Thirdly, migrants are demographically selected; they are characterized by high fertility rates, low mortality rates and are mostly within the reproductive age groups. As such improving their access to SRHR will help correct some of the inequalities that migrants experience due to limited access to SRH services. Furthermore, the study comes just in time as Malawi continues to act on its commitments to the FP2030 agenda to inform decisions on both SRH programming and resource allocation to reach FP2030 goals. Our study also serves as a benchmark for further studies on the impact of migration on SRH outcomes in Malawi and other countries within the region.

This study has limitations, firstly we were unable to draw causal relationships between migration and modern contraceptive use in Malawi due to cross-sectional nature of our study design. However, the strong association found provide evidence that migration has strong impact on modern contraceptive use among women of childbearing age. Secondly, our study did not categorise type of migration involved (rural-rural, urban-rural, rural-urban and urban-urban) to help explain findings better due unavailability of such categories in the datasets. Further research to better understand impact of different migration streams on contraceptive use in Malawi is required. Lastly, MICS datasets are self-reported, making them prone to information bias that could affect our estimates of the association between migration and modern contraceptive.

## Conclusion

We conclude from our findings that migration status has significant impact on modern contraceptives use among women of childbearing age. Our findings highlight the need to find strategies of increasing access to FP by migrating women to correct unequal access to modern FP among migrants. In this way, their SRH needs and rights would be met.

## Data Availability

The data is available on MICS website (https://mics.unicef.org).
